# Supplementary material for: A high affinity switch for cAMP in the HCN pacemaker channels
Source: Nat Commun. 2024 Jan 29;15:843. doi: 10.1038/s41467-024-45136-y (PMC10825183; doi:10.1038/s41467-024-45136-y)
Supplement: Supplementary file 1 — Supplementary Information [file 41467_2024_45136_MOESM1_ESM.pdf]

A high affinity switch for cAMP in the HCN pacemaker channels

Porro et al.

Supplementary Figures and Tables

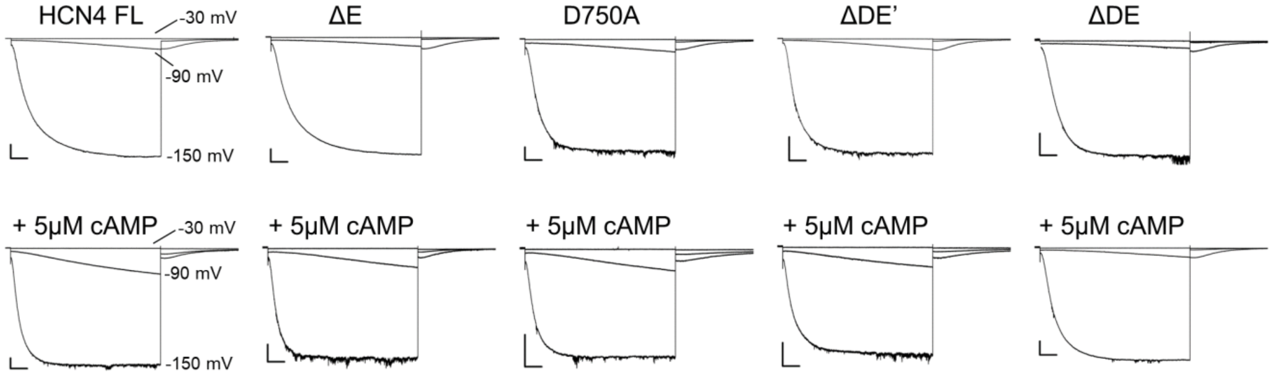

Supplementary Figure 1: Currents recorded from HCN4 constructs.

Whole cell representative current traces recorded at the indicated voltages, from HEK293T cells transiently expressing HCN4 full length (FL), ΔE, D750A, ΔDE' and ΔDE in control solution (top) and with 5 μM cAMP in pipette solution (bottom). Scale bar is 200 pA x 500 ms.

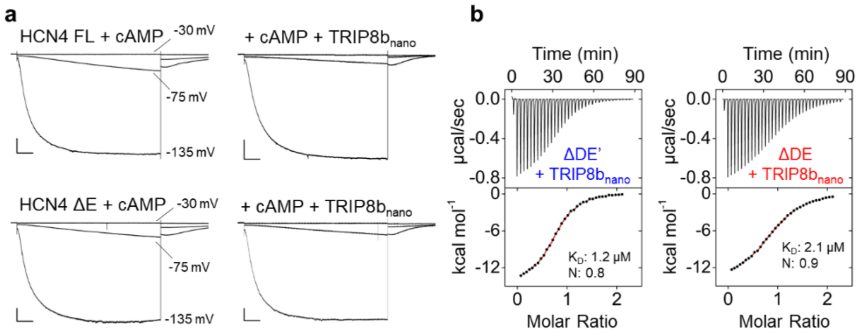

Supplementary Figure 2: HCN4 currents and ITC thermogram with TRIP8bnano

A, Whole cell representative current traces recorded, at the indicated voltages, from HEK293T cells expressing HCN4 full length (FL) and ΔE with cAMP (15 μM for FL and 60 μM for ΔE, left) or with cAMP + 1 μM TRIP8b$_{nano}$ in pipette solution. Scale bar is 200 pA x 500 ms. **B**, examples of ITC thermogram obtained by titrating purified human HCN2 CNBD ΔDE' and ΔDE with TRIP8b$_{nano}$ peptide. Upper panel, heat changes (μcal/sec) during successive injections of TRIP8b peptide. Lower panel, binding curves obtained from data displayed in the upper panel. The peaks were integrated, normalized to TRIP8b peptide concentration and plotted against the molar ratio (TRIP8b$_{nano}$/CNBD). Solid red line represents a nonlinear least-squares fit to a single-site binding model yielding equilibrium dissociation constant (KD) and stoichiometry (N) as reported in Supplementary Table 3.

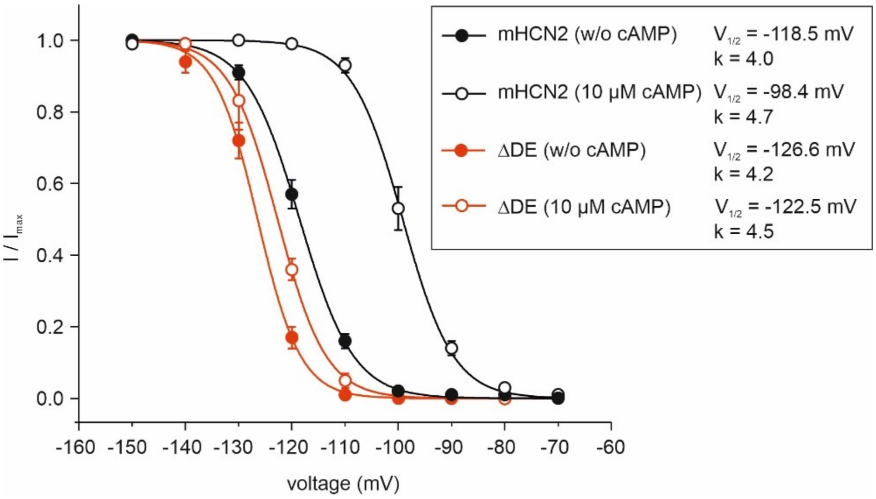

Supplementary Figure 3: Analysis of cAMP response in HCN2 and ΔDE constructs expressed in *Xenopus leavis* oocytes.

Mean-activation curves for mHCN2 and ΔDE channels in inside-out macropatches excised from *Xenopus laevis* ooctyes. mHCN2 (black symbols) and ΔDE (red symbols) currents were measured in control solution (full symbols) and after application of 10 µM cAMP (empty symbols). Half-activation voltage values ($V_{1/2}$) and inverse slope factors (k) are obtained by fitting the Boltzmann equation to the averaged data. Data points represent mean ± SEM (number of recordings ranged from 3 to 8 for each data point).

Supplementary Table 1: List of putative hydrophobic and hydrophilic interactions identified in the D and E helices of cAMP-bound human HCN1 structure (PDB: 6UQF). Bioinformatics tool: Protein/Ligand Interaction Profiler (PLIP). Ref: Salentin, S. et al. PLIP: fully automated protein-ligand interaction profiler. Nucl. Acids Res. (1 July 2015) 43 (W1): W443-W447. doi: 10.1093/nar/gkv315).

Interaction/Pairs	**Location**	**Distance**	**Type**
**L601 – T545**	D helix – P helix	3.7 Å	Hydrophobic Interaction
**L601 – I588**	D helix – C helix	4.0 Å	Hydrophobic Interaction
**L601 – E584**	D helix – C helix	4.1 Å	Hydrophobic Interaction
**L602 – I588**	D helix – C helix	4.1 Å	Hydrophobic Interaction
**K604 – E584**	D helix – C helix	3.3 Å	Salt Bridge
**F605 – I588**	D helix – C helix	3.6 Å	Hydrophobic Interaction
**L622 – R581**	E helix – C helix	4.0 Å	Hydrophobic Interaction
**I625 – V586**	E helix – C helix	3.8 Å	Hydrophobic Interaction
**I625 – L475**	E helix – F' helix	3.7 Å	Hydrophobic Interaction
**V626 – D589**	E helix – C helix	3.6 Å	Hydrophobic Interaction
**D629 – R590**	E helix – C helix	3.3 Å	Salt Bridge
**D629 – R593**	E helix – C helix	3.5 Å	Salt Bridge
**R630 – D589**	E helix – C helix	2.9 Å	Salt Bridge
**V633 – L532**	E helix – β roll	3.8 Å	Hydrophobic Interaction

Supplementary Table 2: Fitting parameters of the activation curves in HCN4, HCN2 and HCN1 channels.

Half-activation voltage ($V_{1/2}$) and inverse slope factor (k) values obtained by fitting experimental data to a Boltzmann function (see Material and Methods) in absence or presence of cAMP, 5 μM for HCN4 (except for numbers preceded by a # where 1 μM was used) and 1 μM for HCN2; n: number of cells tested in each condition; NIE: number of independent experiments (independent experiments are defined as cells measured from independent transfections performed on different days). Values are reported as mean ± SEM. *p values have been calculated by one-side one-way ANOVA with Fisher's test compared to FL HCN4, HCN1 or HCN2; §p values have been calculated by Student's two-tailed unpaired T-test compared to control condition (without cAMP); n.t. not tested.
In HCN1 channels, the shift induced by cAMP was calculated by subtracting the $V_{1/2}$ with the one of the R549E mutation, which is known to reduce the affinity for cAMP by about 1000 times (Chen et al, 2001).

		**$V_{1/2}$ (mV) ± SEM**	**k (mV) ± SEM**	**n**	**NIE**	**$V_{1/2}$ (mV) ± SEM with cAMP**	**k (mV) ± SEM with cAMP**	**n**	**NIE**	**$V_{1/2}$ shift due to cAMP (mV) ± SEM**
**Fig. 1**	**HCN4 FL**	-101.3 ± 1.1	10.8 ± 0.4	9	2	# -89.9 ± 1.6 $^{§p=0.0007}$	11.7 ± 1	7	2	11.4 ± 1.9
	**HCN4 Δc-term**	-101.6 ± 2 $^{*p=0.9}$	11.2 ± 0.6	6	2	# -88.9 ± 1.5 $^{§p<0.0001}$	9.6 ± 0.5	6	2	12.7 ± 2.1 $^{§p=0.65}$
**Fig. 2**	**HCN4 FL**	-102.4 ± 1.3	8.9 ± 0.6	15	5	-87.5 ± 0.7 $^{§p<0.0001}$	10.6 ± 0.4	5	2	14.9 ± 2
	**HCN4 ΔE**	-104.2 ± 1.2 $^{*p=0.02}$	8 ± 0.1	10	3	-97.2 ± 0.6 $^{§p=0.004}$	9.1 ± 1.2	4	1	7 ± 1.9 $^{*p=0.0029}$
	**HCN4 ΔDE'**	-101.8 ± 1 $^{*p=0.7}$	8.3 ± 0.4	10	3	-95.5 ± 0.8 $^{§p=0.007}$	8.4 ± 0.1	3	1	6.3 ± 1.9 $^{*p=0.0029}$
	**HCN4 ΔDE**	-103.8 ± 0.8 $^{*p=0.4}$	7.7 ± 0.5	9	3	-102.4± 0.6 $^{§p=0.2}$	7.6 ± 0.3	7	2	1.4 ± 1 $^{*p<0.0001}$
**Fig. 4**	**HCN4 D750A**	-101.2 ± 0.45 $^{*p=0.4}$	8.4 ± 0.3	8	2	-96 ±1.7 $^{§p=0.001}$	9 ± 0.5	3	1	5.2 ± 1.2 $^{*p=0.001}$
**Fig.5**	**HCN2 FL**	-96.1 ± 0.6	5 ± 0.5	12	4	-89.1 ± 0.7 $^{§p<0.0001}$	4.9 ± 0.3	13	4	7 ± 1.3
	**HCN2 ΔDE**	-98.9 ± 0.9 $^{*p=0.7}$	4 ± 0.4	6	2	-98.4 ± 1.1 $^{§p=0.7}$	4.8 ± 0.1	4	2	0.5 ± 1.4 $^{*p=0.003}$
	**HCN4 ΔE**	-97.8 ± 0.6 $^{*p=0.8}$	4.8 ± 0.5	6	2	-94.8 ± 0.6 $^{§p=0.005}$	4.3 ± 0.2	5	2	3 ± 0.8 $^{*p=0.04}$
	**HCN2 D671A**	-97.3 ± 0.9 $^{*p=0.8}$	3.7 ± 0.7	7	2	-94.5 ± 0.8 $^{§p=0.04}$	4.1 ± 0.3	7	2	2.8 ± 1.2 $^{*p=0.01}$
**Fig.5**	**HCN1 FL**	-73.1 ± 0.4	6.5 ± 0.3	12	3	n.t.				8.3 ± 1
	**HCN1 ΔE**	-78.6 ± 0.8 $^{*p=9E-7}$	5.8 ± 0.9	6	2	n.t.				2.8 ± 1.2 $^{*p=0.001}$
	**HCN1 D629A**	-77.5 ± 0.6 $^{*p=3E-6}$	5.7 ± 0.3	10	3	n.t.				3.9 ± 1 $^{*p=0.002}$
	**HCN1 R549E**	-81.4 ± 0.9 $^{*p=7E-9}$	6.5 ± 0.8	4	1	n.t.				

Supplementary Table 3: Equilibrium dissociation constant (K_D) and stoichiometry (N) derived from ITC experiments. Values are reported as mean ± SEM. *p values have been calculated by one-side one-way ANOVA with Fisher's test (ΔE, ΔDE' and ΔDE were compared to ΔC-term; D698A and R662A were compared to ΔE; L670G - L671G and L670G - L671G - D698A were compared to ΔDE)

HCN2 CNBD (titrated) (20 μM)	**Titrant**	**K_D (μM)**	**N**	**Number of indipendent experiments**
**ΔC-term**	cAMP (200 μM)	0.3 ± 0.01	0.4 ± 0.1	3
**ΔE**	cAMP (200 μM)	1.2 ± 0.1 $^{p=0.001}$	1.0 ± 0.1	3
**ΔDE'**	cAMP (200 μM)	1.2 ± 0.03 $^{p=0.001}$	0.8 ± 0.1	3
**ΔDE**	cAMP (500 μM)	9.5 ± 0.7 $^{p=7E-17}$	0.4 ± 0.1	3
**D698A**	cAMP (200 μM)	1.5 ± 0.1 $^{p=0.24}$	0.8 ± 0.1	3
**R662A**	cAMP (200 μM)	1 ± 0.03 $^{p=0.48}$	0.9 ± 0.1	3
**L670G - L671G**	cAMP (400 μM)	3 ± 0.1 $^{p=1.8E-14}$	1.1 ± 0.1	3
**L670G - L671G - D698A**	cAMP (400 μM)	6.7 ± 0.2 $^{p=9E-9}$	0.9 ± 0.1	3
**ΔC-term**	$Trip8b_{nano}$ (200 μM)	0.6 ± 0.05	0.4 ± 0.1	3
**ΔE**	$Trip8b_{nano}$ (200 μM)	0.7 ± 0.01 $^{p=0.15}$	1.0 ± 0.1	3
**ΔDE'**	$Trip8b_{nano}$ (200 μM)	1.2 ± 0.03 $^{p=2E-5}$	0.8 ± 0.1	3
**ΔDE**	$Trip8b_{nano}$ (200 μM)	2.1 ± 0.07 $^{p=9E-5}$	0.9 ± 0.1	3

Supplementary Table 4: Fitting parameters of the activation curves in HCN4 channel with TRIP8b$_{nano}$.

Half-activation voltage ($V_{1/2}$) and inverse slope factor (k) values obtained by fitting experimental data to a Boltzmann function (see Material and Methods) in control solution, with cAMP (15 µM for HCN4 FL and 60 µM for ΔE) and with 1µM TRIP8b$_{nano}$ in pipette solution; n= number of cells tested in each condition. Values are reported as mean ± SEM. NIE: number of independent experiments (independent experiments are defined as cells measured from independent transfections performed on different days).

§p values have been calculated by two-tailed unpaired Student's T-test compared to control condition (without cAMP); *p values have been calculated by two-tailed unpaired Student's T-test compared to "+ cAMP".

	$V_{1/2}$ (mV)	k (mV)	n	NIE	$V_{1/2}$ (mV) + cAMP	k (mV) + cAMP	n	$V_{1/2}$ (mV) + cAMP + TRIP8b$_{nano}$	k (mV) + cAMP + TRIP8b$_{nano}$	n	NIE	$V_{1/2}$ shift due to TRIP8b$_{nano}$ (mV)
**HCN4 FL**	-98.8 ± 0.4	8.1 ± 0.6	8	2	-79.3 ± 0.9 $^{§p<0.0001}$	10.2 ± 1	9	-86 ± 0.7 $^{*p<0.0001}$	9.8 ± 0.9	10	2	-6.7 ± 1.1
**HCN4 ΔE**	-98.3 ± 0.5 $^{§p=0.45}$	8.1 ± 0.8	5	2	-81.1 ± 0.8 $^{§p<0.0001}$	9.8 ± 0.8	7	-87.8 ± 0.8 $^{*p<0.0001}$	9 ± 1.1	7	2	-6.8 ± 1.3
