# Supplementary material for: A high affinity switch for cAMP in the HCN pacemaker channels
Source: Nat Commun. 2024 Jan 29;15:843. doi: 10.1038/s41467-024-45136-y (PMC10825183; doi:10.1038/s41467-024-45136-y)
Supplement: Supplementary file 3 — Reporting Summary [file 41467_2024_45136_MOESM3_ESM.pdf]

Corresponding author(s): Anna Moroni

Last updated by author(s): 2023_08_29

Reporting Summary

Nature Portfolio wishes to improve the reproducibility of the work that we publish. This form provides structure for consistency and transparency in reporting. For further information on Nature Portfolio policies, see our Editorial Policies and the Editorial Policy Checklist.

Statistics

For all statistical analyses, confirm that the following items are present in the figure legend, table legend, main text, or Methods section.

n/a	Confirmed	
☐	☒	The exact sample size (*n*) for each experimental group/condition, given as a discrete number and unit of measurement
☐	☒	A statement on whether measurements were taken from distinct samples or whether the same sample was measured repeatedly
☐	☒	The statistical test(s) used AND whether they are one- or two-sided<br>*Only common tests should be described solely by name; describe more complex techniques in the Methods section.*
☒	☐	A description of all covariates tested
☒	☐	A description of any assumptions or corrections, such as tests of normality and adjustment for multiple comparisons
☐	☒	A full description of the statistical parameters including central tendency (e.g. means) or other basic estimates (e.g. regression coefficient) AND variation (e.g. standard deviation) or associated estimates of uncertainty (e.g. confidence intervals)
☐	☒	For null hypothesis testing, the test statistic (e.g. *F*, *t*, *r*) with confidence intervals, effect sizes, degrees of freedom and *P* value noted<br>*Give P values as exact values whenever suitable.*
☒	☐	For Bayesian analysis, information on the choice of priors and Markov chain Monte Carlo settings
☒	☐	For hierarchical and complex designs, identification of the appropriate level for tests and full reporting of outcomes
☒	☐	Estimates of effect sizes (e.g. Cohen's *d*, Pearson's *r*), indicating how they were calculated

Our web collection on statistics for biologists contains articles on many of the points above.

Software and code

Policy information about availability of computer code

Data collection

Figures 1,2,4,5,7, S1, S2: All the patch clamp data in HEK cells were collected using Clampex 10.7 software, belonging to the pClamp 10 software suite (Molecular Devices, CA, USA) or EZPatch 2.3.2 (Elements srl, Cesena, Italy).

Figures 2,4,6,7,S2: ITC data were collected using MicroCal Origin software 7 (OriginLab, Northampton, MA, USA).

Figure 3: cAMP content assay data were collected using UNICORN 5.1 (Cytivalifesciences, Marlborough, MA, USA)

Figure 8: Patch clamp fluormetry recordings were performed with an LSM 710 confocal microscope (Carl Zeiss, Jena, Germany) and were triggered by the ISO3 software (MFK, Niedernhausen, Germany).

Data analysis

Figures 1,2,4,5,7,S1, S2: All the patch clamp data in HEK cells were analyzed using Clampfit 10.7, belonging to the pClamp 10 software suite (Molecular Devices, CA, USA) and with OriginPRO 2022 (OriginLab, Northampton, MA, USA). Statistical analysis was perfomed OriginPRO 2022 (OriginLab, Northampton, MA, USA, USA)

Figures 2,4,6,7, S2: ITC data were analyzed with MicroCal Origin software 7 (OriginLab, Northampton, MA, USA). Statistical analysis was perfomed OriginPRO 2022 (OriginLab, Northampton, MA, USA, USA)

Figure 3: cAMP content assay data were analyzed with with OriginPro9.0G (OriginLab, Northampton, MA, USA). Statistical analysis was perfomed OriginPro9.0G (OriginLab, Northampton, MA, USA, USA).

Figure 8: Patch clamp fluorometry data were analyzed with with OriginPRO 2022 (OriginLab, Northampton, MA, USA). Statistical analysis was perfomed OriginPRO 2022 (OriginLab, Northampton, MA, USA, USA)

For manuscripts utilizing custom algorithms or software that are central to the research but not yet described in published literature, software must be made available to editors and reviewers. We strongly encourage code deposition in a community repository (e.g. GitHub). See the Nature Portfolio guidelines for submitting code & software for further information.

Data

Policy information about availability of data

All manuscripts must include a data availability statement. This statement should provide the following information, where applicable:

- Accession codes, unique identifiers, or web links for publicly available datasets
- A description of any restrictions on data availability
- For clinical datasets or third party data, please ensure that the statement adheres to our policy

The authors declare that the data supporting the findings of this study are available within the article and its supplementary information files, and from the corresponding author on request. A reporting summary for this article is available as a supplementary information file. The source data underlying Figures and Supplementary Figures are provided as a Source Data file.
Previously published PDB codes are: 3U10 [https://www.rcsb.org/structure/3u10]; 5U6O [https://www.rcsb.org/structure/5U6O]; 5U6P [https://www.rcsb.org/structure/5U6P]; 6UQF https://www.rcsb.org/structure/6UQF].

Research involving human participants, their data, or biological material

Policy information about studies with human participants or human data. See also policy information about sex, gender (identity/presentation), and sexual orientation and race, ethnicity and racism.

Reporting on sex and gender	N/A
Reporting on race, ethnicity, or other socially relevant groupings	N/A
Population characteristics	N/A
Recruitment	N/A
Ethics oversight	N/A

Note that full information on the approval of the study protocol must also be provided in the manuscript.

Field-specific reporting

Please select the one below that is the best fit for your research. If you are not sure, read the appropriate sections before making your selection.

☒ Life sciences ☐ Behavioural & social sciences ☐ Ecological, evolutionary & environmental sciences

For a reference copy of the document with all sections, see nature.com/documents/nr-reporting-summary-flat.pdf

Life sciences study design

All studies must disclose on these points even when the disclosure is negative.

Sample size	For patch clamp experiments in HEK cells and for PCF recordings in Xenopus laevis oocytes, we use as a sample size, usually between 3-10 cells for each condition. These numbers are adequate for the kind of measurements, based on the fact that the signal to noise ratio is very high (at least >4).
Data exclusions	Patch Clamp experiments in HEK cells: Cells with an access resistance (Rs) larger than 20 MOhm and a Seal Resistance lower than 1 GOhm were excluded by the mean.<br>ITC experiments: no data were excluded by the mean.<br>PCF recordings: no data were excluded by the mean.
Replication	Patch clamp experiments on cell cultures were performed once or twice in a week, cells were patched 12-24h after (transient) transfection. Each condition/protocol was tested at least in 3 independent experiments, each time the number of cells tested was > 3.<br>ITC experiments: at least 3 independent experiments were made for each condition.<br>PCF recordings: at least 3 independent experiments were made for each condition.<br>In all cases we confirm that all attempt at replication were successfull.
Randomization	Not relevant in the type of experiments presented in this work as the operator cannot influence the outcome of the measurement
Blinding	Not relevant in the type of experiments presented in this work as the operator cannot influence the outcome of the measurement

April 2023

Reporting for specific materials, systems and methods

We require information from authors about some types of materials, experimental systems and methods used in many studies. Here, indicate whether each material, system or method listed is relevant to your study. If you are not sure if a list item applies to your research, read the appropriate section before selecting a response.

Materials & experimental systems

n/a	Involved in the study
☒	☐ Antibodies
☐	☒ Eukaryotic cell lines
☒	☐ Palaeontology and archaeology
☐	☒ Animals and other organisms
☒	☐ Clinical data
☒	☐ Dual use research of concern
☒	☐ Plants

Methods

n/a	Involved in the study
☒	☐ ChIP-seq
☒	☐ Flow cytometry
☒	☐ MRI-based neuroimaging

Eukaryotic cell lines

Policy information about cell lines and Sex and Gender in Research

Cell line source(s)	HEK 293 T: purchased from ATCC CRL-11268™.
Authentication	HEK 293 T cells were authenticated by ATCC by PCR assys
Mycoplasma contamination	HEK293T cell were periodically tested (once a month) for Mycomplasma contamination using MycoAlter detection kit (Lonza) and resulted always negative.
Commonly misidentified lines (See ICLAC register)	HEK293 cells (but not HEK293T) are listed in the ICLAC database for possible contamination by HeLa cells. We think that for our purposes, i.e. heterologous expression of HCN channels, such a contamination should not matter.

Animals and other research organisms

Policy information about studies involving animals; ARRIVE guidelines recommended for reporting animal research, and Sex and Gender in Research

Laboratory animals	Female African claw frogs (Xenopus laevis) were used to harvest oocytes as heterologous cell system. The frogs were between 2 and 3 years old. They were purchased from Xenopus1 (USA).
Wild animals	The study did not involve wild animals.
Reporting on sex	Female African claw frogs (Xenopus laevis) were used.
Field-collected samples	The study did not involve samples collected from the field.
Ethics oversight	The surgery procedures were carried out in accordance with the German Animal Welfare Act with the approval of the Thuringian State Office for Consumer Protection on 30.08.2013 and 09.05.2018.

Note that full information on the approval of the study protocol must also be provided in the manuscript.
